# Use of Glucagon‐Like Peptide‐1 Receptor Agonists and Risk of Parkinson's Disease: Scandinavian Cohort Study

**DOI:** 10.1111/dom.70760

**Published:** 2026-04-17

**Authors:** Arvid Engström, Henrik Svanström, Anders Hviid, Björn Eliasson, Soffia Gudbjörnsdottir, Viktor Wintzell, Kristian Hveem, Christian Jonasson, Mads Melbye, Peter Ueda, Björn Pasternak

**Affiliations:** ^1^ Clinical Epidemiology Division, Department of Medicine, Centre for Pharmacoepidemiology, Solna Karolinska Institutet Stockholm Sweden; ^2^ Department of Epidemiology Research Statens Serum Institut Copenhagen Denmark; ^3^ Department of Drug Design and Pharmacology, Faculty of Health and Medical Sciences, Pharmacovigilance Research Center University of Copenhagen Copenhagen Denmark; ^4^ Department of Medicine Sahlgrenska University Hospital Gothenburg Sweden; ^5^ The Swedish National Diabetes Register Vastra Gotalandsregionen Gothenburg Sweden; ^6^ Department of Molecular and Clinical Medicine, Institute of Medicine University of Gothenburg Gothenburg Sweden; ^7^ Department of Public Health and Nursing, Faculty of Medicine and Health Science, HUNT Center for Molecular and Clinical Epidemiology NTNU—Norwegian University of Science and Technology Trondheim Norway; ^8^ Faculty of Medicine, HUNT Research Center NTNU—Norwegian University of Science and Technology Levanger Norway; ^9^ Department of Clinical Medicine University of Copenhagen Copenhagen Denmark; ^10^ Department of Pediatrics Stanford University School of Medicine Stanford California USA; ^11^ Danish Cancer Institute Copenhagen Denmark

**Keywords:** disease prevention, GLP‐1 analogue, observational study, pharmaco‐epidemiology

## Abstract

**Aims:**

To investigate the association between use of GLP‐1 receptor agonists and incident Parkinson's disease.

**Material and Methods:**

Cohort study using data from nationwide registers in Denmark, Norway and Sweden and an active‐comparator, new‐user design. We included 158 961 new users of GLP‐1 receptor agonists and 188 065 new users of sulfonylureas, aged 45 years or older. Liraglutide accounted for 72.9% of GLP‐1 receptor agonist follow‐up time, followed by semaglutide (13.4%), exenatide (7.3%), dulaglutide (5.1%) and lixisenatide (1.3%). The primary outcome was incident Parkinson's disease, defined as a first‐ever diagnosis of Parkinson's disease (ICD‐10 G20) or Parkinson's disease dementia (ICD‐10 F02.3) in national patient registers. Cox regression with propensity score weighting was used to estimate hazard ratios (HRs) and control for confounding.

**Results:**

Mean age was 65 years and 43% were female. Incidence rates for Parkinson's disease were 5.2 and 8.0 per 10 000 person‐years among GLP‐1 receptor agonist and sulfonylurea users, respectively (adjusted HR 0.81 [95% CI 0.68–0.96]). Results were consistent in a 2‐year lag‐time analysis (HR 0.84 [95% CI 0.70–1.02]) after excluding or censoring users of DPP‐4 inhibitors at cohort entry or during follow‐up (HR 0.74 [95% CI 0.60–0.93]) and in subgroup analyses by sex and age.

**Conclusion:**

In this large observational cohort study, use of GLP‐1 receptor agonists compared with sulfonylureas was associated with a lower risk of incident Parkinson's disease. These findings support a potential neuroprotective role of GLP‐1 receptor agonists, though replication in additional studies is needed.

## 1 Introduction

Parkinson's disease is a progressive neurodegenerative disorder. Type 2 diabetes has been associated with an increased risk of Parkinson's disease as well as faster disease progression [1, 2, 3]. There are no curative or disease‐modifying therapies available for Parkinson's disease for clinical use. Identifying potential interventions that may prevent the disease is therefore of high priority [4]. Shared pathophysiological features exist between type 2 diabetes and Parkinson's disease [3, 5].

Glucagon‐like peptide‐1 (GLP‐1) receptor agonists are glucose‐lowering drugs commonly prescribed for type 2 diabetes. Growing evidence suggests that GLP‐1 receptor agonists modulate pathophysiological mechanisms in the brain of relevance for the development of Parkinson's disease, including neuroinflammation, mitochondrial dysfunction, amyloid aggregation and insulin signalling [6, 7, 8, 9].

In support of the hypothesis that GLP‐1 receptor agonists may provide benefits to patients with Parkinson's disease, a randomized controlled trial of 62 patients with Parkinson's disease showed that treatment with the GLP‐1 receptor agonist exenatide had a positive effect on motor function in comparison to placebo [10]. Moreover, a randomized controlled trial including 156 patients with early Parkinson's disease showed slower progression of motor symptoms in patients treated with the GLP‐1 receptor agonist lixisenatide compared to placebo [11].

Whether GLP‐1 receptor agonists reduce the risk of incident Parkinson's disease remains uncertain. No clinical trials have addressed this question. Observational studies report a lower risk among GLP‐1RA users, but limited outcome events and methodological limitations preclude firm conclusions [12, 13, 14].

We aimed to examine the association between use of GLP‐1 receptor agonists and the risk of incident Parkinson's disease.

## 2 Methods

### 2.1 Data Sources

We used nationwide health and administrative registers in Denmark, Norway and Sweden including population registers (vital status, demographics), patient registers (comorbidities, outcomes), prescribed drug registers (study drugs, co‐medications), Statistics Denmark and Statistics Sweden (socioeconomic variables) and the Swedish National Diabetes Register (glycated haemoglobin, blood pressure, albuminuria, estimated glomerular filtration rate [eGFR], body mass index and smoking). Data sources are described in detail in the Supporting Information.

### 2.2 Study Design and Study Population

We conducted an active‐comparator new‐user cohort study [15]. We included patients, aged 45 years or older, who initiated a GLP‐1 receptor agonist or the comparator, sulfonylureas, between January 1st, 2007, and December 31st, 2021, in Denmark and Sweden; and between January 1st, 2010, and December 31st, 2018, in Norway. New use was defined as no use of a GLP‐1 receptor agonist at any time before cohort entry or a sulfonylurea 1 year before cohort entry. This allowed patients to contribute separate treatment episodes, meaning they could enter the cohort first upon initiating a sulfonylurea and later upon initiating a GLP‐1 receptor agonist. The anatomic therapeutic chemical codes for the study drugs are provided in Table S1. The date of filling the first prescription constituted cohort entry.

We excluded patients who had a history of Parkinson's disease, secondary parkinsonism and Lewy body dementia at any time before cohort entry and patients who used any anti‐Parkinson drug within the last year before cohort entry. Further exclusion criteria were history of dialysis or renal transplantation, end stage illness including dementia, drug misuse, severe pancreatic disorders and use of liraglutide with obesity indication at any time before cohort entry, neither use of any prescription drug nor any specialist care contact in the previous year and hospital admission for any reason within 30 days before cohort entry (Table S2).

### 2.3 Outcome

The outcome was a first diagnosis of Parkinson's disease (ICD‐10 code: G20) or dementia in Parkinson's disease (ICD‐10 code: F02.3), registered as diagnoses in the patient registers during any type of hospital contact (hospitalization or outpatient visit; primary or secondary diagnosis).

### 2.4 Follow Up

Patients were followed from treatment initiation until the study outcome or until they were censored due to emigration, death, end of the study period, or a switch in therapy (initiation of a sulfonylurea among patients who entered the study on a GLP‐1 receptor agonist and vice versa).

### 2.5 Statistical Analyses

We used standardized mortality ratio (SMR) weighting based on propensity score to adjust for confounding, estimating the average treatment effect among the treated [16]. The probability of starting a GLP‐1 receptor agonist versus a sulfonylurea was estimated using a logistic regression model containing 54 variables, as registered at cohort entry. The variables comprised sociodemographic characteristics, diabetes complications, co‐morbidities, antidiabetic medications, non‐diabetes medications and measures of co‐morbidity burden, frailty and healthcare utilization (Table S3). The propensity score was estimated in each country separately and patients with propensity scores outside of the common range of the propensity score distribution were excluded. Analyses were performed in a pooled dataset from the three countries. To limit the influence of extreme propensity score weights, we applied weight truncation at the 1st and 99th percentile of the weight distribution [17]. The covariate balance after weighting was assessed with standardized difference; differences below 10% were considered as good balance.

A Cox proportional hazards regression model with time since start of treatment as the time scale was used to estimate hazard ratios (HRs) [16]. A 95% confidence interval (CIs) that did not overlap 1 was considered as a statistically significant difference. We described the cumulative incidence using Kaplan–Meier curves. The reported incidence rates are unadjusted. Absolute incidence rate differences were calculated using Poisson regression with an identity link function. Additional analyses were conducted to assess incidence rate differences at prespecified time intervals (3, 5 and 7 years after treatment initiation).

We conducted prespecified subgroup analyses by age group (45–69 and ≥ 70 years) and sex. A separate propensity score was estimated within each subgroup. Effect modification by subgroup status was examined by including an interaction term between treatment status and subgroup in the Cox model; in these analyses, *p*‐values of < 0.05 were considered statistically significant. We also conducted analyses by country.

We conducted several prespecified sensitivity analyses. First, as Parkinson's disease may develop slowly before becoming manifest and diagnosed [18, 19], we performed an analysis using a lag‐time period of 2 years between treatment initiation and the start of follow‐up. Second, we restricted the outcome definition to primary diagnoses in the patient registers. Third, to address the temporal treatment trends of increased GLP‐1 agonist use and decreased sulfonylurea use during the study period we conducted two complementary analyses: (1) a propensity score model incorporating calendar time as a covariate, and (2) a calendar time‐specific propensity score analysis in which we stratified the study period into biennial calendar intervals and estimated separate propensity scores within each time stratum. Fourth, since both GLP‐1 receptor agonists and dipeptidyl peptidase‐4 (DPP‐4) inhibitors target the incretin system and it has been suggested that DPP‐4 inhibitors may have a beneficial effect in Parkinson's disease [20], we performed an analysis in which patients with use of DPP‐4 inhibitors at any time before cohort entry were excluded and patients who initiated DPP‐4 inhibitors during follow‐up were censored. Fifth, to address potential residual confounding by unmeasured frailty, or other age‐related factors that may not be fully captured by the covariates included in the propensity score model, we performed a sensitivity analysis excluding participants aged > 80 years. Sixth, to evaluate the robustness of our primary asymmetric lookback approach, we conducted a sensitivity analysis applying a strict new‐user definition that required no prior exposure to either study drug class at any time before cohort entry. Within this cohort, we also performed an analysis employing an intention‐to‐treat exposure definition, where patients were not censored at treatment switch. Seventh, we performed an inverse probability of censoring weighting analysis to account for potential informative censoring. Eighth, to assess outcome validity, we conducted a sensitivity analysis in which the outcome was defined as a Parkinson's disease diagnosis recorded at two separate visits. Ninth, to account for the competing risk of death, a sensitivity analysis was performed using a Fine and Gray proportional subdistribution hazards model with death from any cause treated as a competing event. Finally, in the Swedish part of the cohort we expanded the propensity score to include additional variables including glycated haemoglobin, blood pressure, albuminuria, eGFR, body mass index and smoking. Given the proportion of missing values for the additional variables (Table S4), multiple imputation (fully conditional specification imputation) with 10 imputed datasets was used [21]. Imputation was based on all variables included in the propensity score, the additional variables and the outcome variable.

The study was approved by the Regional Ethics Committee in Stockholm, Sweden, and the Regional Committee for Medical and Health Research Ethics, Norway. In Denmark, approval by an ethics committee is not required for register‐based research.

## 3 Results

### 3.1 Study Population

**FIGURE 1:**
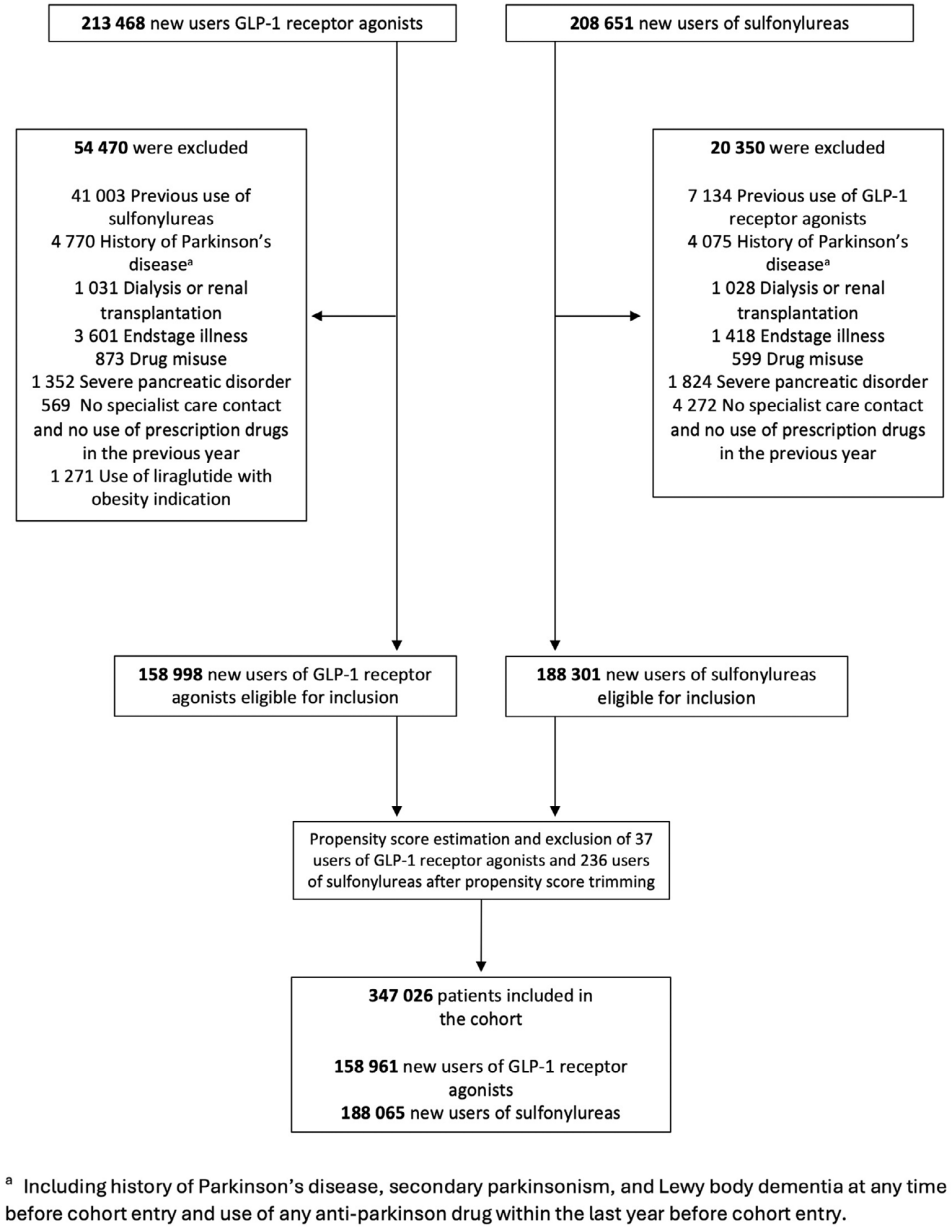
Flow chart of patient inclusion in the study cohort, Sweden, Denmark and Norway.

Our analysis included 158 961 eligible patients initiating GLP‐1 receptor agonists and 188 065 initiating sulfonylureas, providing 559 979 and 1 156 732 patient‐years of follow‐up, respectively (Figure 1).  Population characteristics before and after propensity score weighting are shown in Table 1; covariates in the two groups were well‐balanced after weighting. Mean age of the study population was 65 years and 43% were female. Median follow‐up time was 2.5 years (interquartile range 1.0–5.2) for GLP‐1 receptor agonist initiators and 5.8 years (2.9–9.0) for sulfonylurea initiators. The proportion of total follow‐up time by type of GLP‐1 receptor agonist was 72.9% for liraglutide, 13.4% for semaglutide, 7.3% for exenatide, 5.1% for dulaglutide, 1.3% for lixisenatide.

**TABLE 1:** Patient characteristics at cohort entry for users of GLP‐1 receptor agonists and sulfonylureas before and after propensity score weighting.

	Unweighted *n* (%)			Propensity score weighted %		
	GLP‐1 receptor agonists (*N* = 158 961)	Sulfonylureas (*N* = 188 065)	Standardized difference (%)	GLP‐1 receptor agonists	Sulfonylureas	Standardized difference (%)
Male	90 992 (57)	108 090 (57)	0.5	57	57	1.0
Age, mean (SD) in years	63 (10)	67 (11)	—	63 (10)	62 (9)	
Age group in years						
45–49	16 314 (10)	14 114 (8)	9.7	10	11	3.7
50–54	22 533 (14)	18 802 (10)	12.8	14	15	2.0
55–59	26 199 (16)	23 481 (12)	11.4	16	17	1.7
60–64	27 368 (17)	28 901 (15)	5.0	17	17	0.5
65–69	25 441 (16)	30 088 (16)	0.0	16	16	0.8
70–74	21 143 (13)	26 422 (14)	2.2	13	12	2.8
75–79	12 923 (8)	20 555 (11)	9.5	8	7	3.0
80–84	5103 (3)	14 688 (8)	20.3	3	3	1.0
≥ 85	1937 (1)	11 014 (6)	25.3	1	1	1.0
Place of birth						
Scandinavia	136 653 (86)	156 009 (83)	8.3	86	85	3.4
Rest of Europe	9982 (6)	13 266 (7)	3.1	6	7	1.7
Outside Europe	12 326 (8)	18 790 (10)	7.9	8	9	2.9
Civil status						
Married/living with partner	88 851 (56)	102 683 (55)	2.6	56	56	0.4
Single	69 856 (44)	84 142 (45)	1.6	44	44	0.3
Education^a^						
Primary‐/secondary school· vocational training	112 607 (71)	126 598 (67)	7.6	71	70	1.6
Short tertiary education	11 439 (7)	9679 (5)	8.5	7	7	0.4
Medium or long tertiary education	19 757 (12)	15 116 (8)	14.5	12	13	1.5
Missing	2726 (2)	6625 (4)	28.6	10	10	0.5
Year of cohort entry^b^						
2007–09	2411 (2)	50 344 (27)	—	2	22	—
2010–11	12 449 (8)	40 765 (22)	—	8	19	—
2012–13	12 272 (8)	31 990 (17)	—	8	15	—
2014–15	13 728 (9)	26 867 (14)	—	9	14	—
2016–17	21 217 (13)	21 250 (11)	—	13	13	—
2018–19	35 784 (23)	10 470 (6)	—	23	8	—
2020–21	61 100 (38)	6379 (3)	—	38	7	—
Comorbidities						
Ischemic heart disease	24 967 (16)	28 625 (15)	1.3	16	15	3.1
Heart failure/cardiomyopathy	10 768 (7)	12 757 (7)	0.0	7	6	3.1
Stroke/cerebrovascular disease	7243 (5)	10 861 (6)	5.5	5	4	1.1
Head trauma	1755 (1)	2422 (1)	1.7	1	1	0.0
Other neurologic disease	26 707 (17)	19 543 (10)	18.8	17	16	1.8
Arrythmia	16 458 (10)	20 222 (11)	1.3	10	10	2.7
Peripheral arterial disease (incl. amputation)	8369 (5)	8303 (4)	4.0	5	5	3.0
Kidney disease	16 521 (10)	12 245 (7)	14.0	10	9	3.4
Diabetes complications	38 890 (24)	29 244 (16)	22.4	24	22	5.9
COPD	6414 (4)	7911 (4)	0.9	4	4	1.0
Other lung disease	10 618 (7)	9500 (5)	6.9	7	6	1.3
Venous thromboembolism	3593 (2)	3576 (2)	2.5	2	2	0.2
Cancer (excl. non‐melanoma skin cancer)	9959 (6)	14 099 (7)	4.9	6	6	1.0
Melanoma	667 (0)	672 (0)	1.0	0	0	0.1
Liver disease	3174 (2)	2748 (1)	4.1	2	2	0.5
Thyroid disease	2167 (1)	1715 (1)	4.3	1	1	0.5
Osteoporosis	3650 (2)	5606 (3)	4.3	2	2	0.7
Fracture in the previous year	3060 (2)	4332 (2)	2.6	2	2	0.2
Alcohol‐related disorders	2577 (2)	3040 (2)	0.0	2	2	0.6
Health care utilization in previous year						
Hospitalization due to neurological causes	1422 (1)	1619 (1)	0.4	1	1	0.6
Hospitalization due to other causes	36 181 (23)	43 832 (23)	1.3	23	22	1.9
Outpatient hospital contact due to neurological causes	7143 (4)	5240 (3)	9.1	4	4	0.6
Outpatient hospital contact due to other causes	106 582 (67)	107 202 (57)	20.8	67	66	1.9
Diabetes drugs in previous 6 months						
Metformin	120 226 (76)	138 504 (74)	4.6	76	78	5.2
Any 2nd line anti‐diabetic	106 776 (67)	40 595 (22)	103.3	67	68	1.1
Prescription drug use in previous year						
ACE‐inhibitors or ARB	111 618 (70)	111 557 (59)	23.0	70	69	2.8
Calcium‐channel blocker	55 459 (35)	54 042 (29)	13.2	35	34	2.9
Spironolactone	10 264 (6)	9594 (5)	5.8	6	6	2.4
Loop diuretic	29 009 (18)	32 910 (17)	2.0	18	17	4.0
Other diuretic	30 979 (19)	36 108 (19)	0.7	19	18	2.9
Beta‐blocker	60 286 (38)	70 660 (38)	0.7	38	36	3.5
Other cardiovascular drugs	15 215 (10)	23 663 (13)	9.6	10	9	1.8
Antiarrhythmic drugs	1087 (1)	1146 (1)	0.9	1	1	0.5
Platelet inhibitors	54 265 (34)	71 996 (38)	8.6	34	32	3.6
Anticoagulants	16 681 (10)	16 575 (9)	5.7	10	10	2.5
Lipid lowering drug	115 663 (73)	112 831 (60)	27.3	73	71	3.4
Lithium	630 (0)	706 (0)	0.3	0	0	0.0
Antidepressants	30 102 (19)	28 920 (15)	9.5	19	19	0.4
Typical antipsychotics	2022 (1)	3639 (2)	5.3	1	1	0.1
Atypical antipsychotics	3962 (2)	3906 (2)	2.8	2	3	0.4
Anxiolytic, hypnotic or sedative	27 754 (17)	40 346 (21)	10.1	17	18	0.3
Beta‐2 agonist inhalant	17 948 (11)	15 924 (8)	9.5	11	11	0.4
Anticholinergic inhalant	5311 (3)	6809 (4)	1.5	3	3	0.0
Glucocorticoid inhalant	17 668 (11)	17 681 (9)	5.6	11	11	0.6
Oral glucocorticoid	13 613 (9)	18 159 (10)	3.8	9	9	0.1
Opioid	30 708 (19)	34 589 (18)	2.4	19	19	0.2
Antiepileptic	8356 (5)	6232 (3)	9.6	5	5	0.8
Urate‐lowering drug	9920 (6)	10 117 (5)	3.7	6	6	1.4
No. of prescription drugs in last year						
0–5	17 792 (11)	39 769 (21)	27.3	11	12	2.4
6–10	61 643 (39)	79 697 (42)	7.3	39	40	2.4
11–15	47 799 (30)	44 135 (23)	15.0	30	29	1.6
> 15	31 727 (20)	24 464 (13)	18.8	20	19	3.1

Abbreviations: ACE, angiotensin converting enzyme; ARB, angiotensin receptor blocker; ARNI, angiotensin receptor neprilysin inhibitor; COPD, chronic obstructive pulmonary disease.

^a^ Information about education is not available in Norway.

^b^ Not included in the propensity score.

### 3.2 Primary Analysis

**FIGURE 2:**
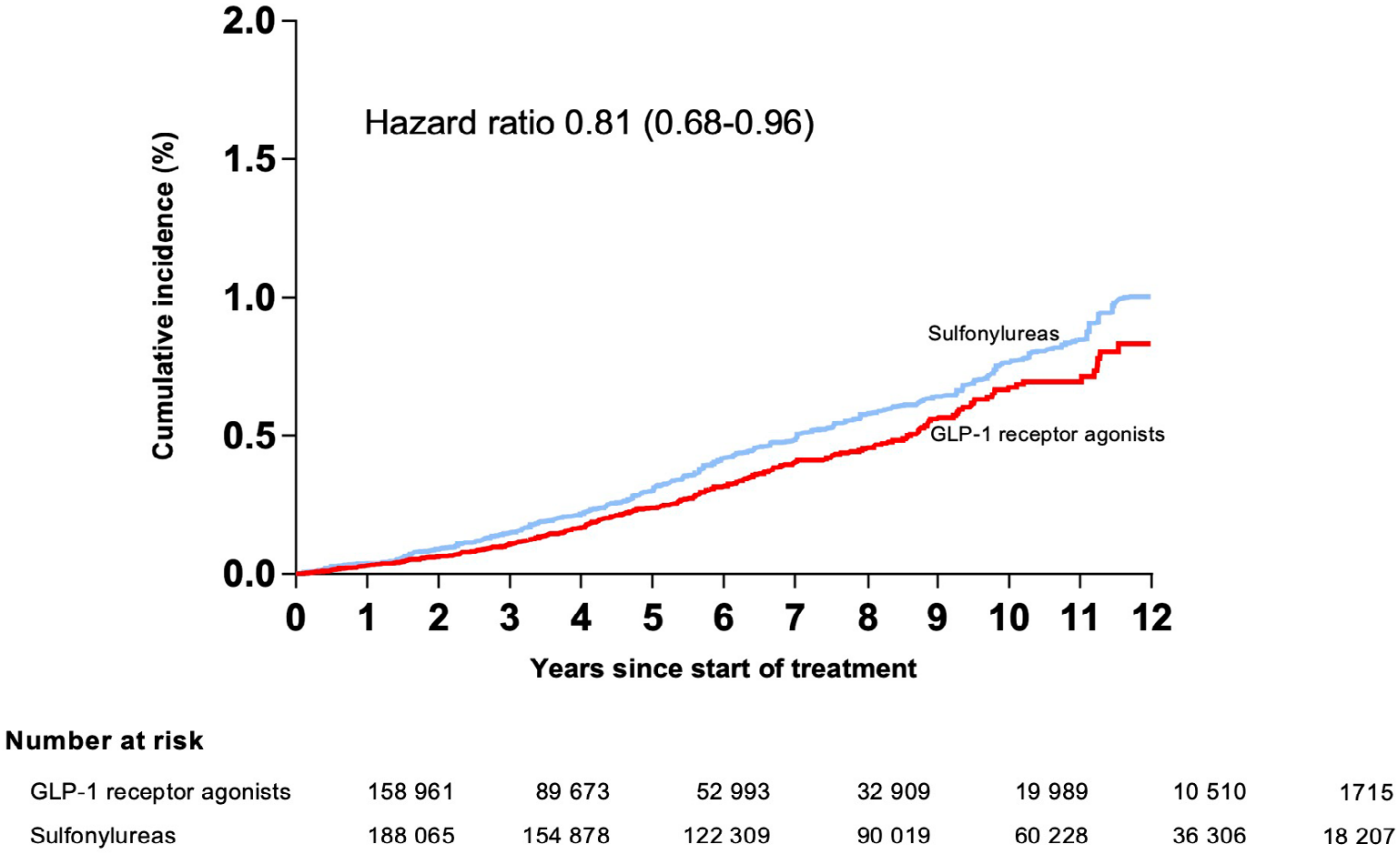
Adjusted cumulative incidence of incident Parkinson's disease among users of GLP‐1 receptor agonists compared with users of sulfonylureas.

Figure 2 shows the adjusted cumulative incidence of Parkinson's disease. During follow‐up, 290 users of GLP‐1 receptor agonists and 927 users of sulfonylureas received a first diagnosis of Parkinson's disease. The incidence rate for incident Parkinson's disease was 5.2 per 10 000 person‐years with use of GLP‐1 receptor agonists and 8.0 per 10 000 person‐years with use of sulfonylureas. The unadjusted hazard ratio (HR) was 0.73 (95% CI 0.64–0.83) and the adjusted HR was 0.81 (95% CI 0.68–0.96). The absolute rate difference was −2.1 events per 10 000 person‐years (95% CI −3.2 to −1.0). Time‐specific incidence rate differences are presented in Table S5.

### 3.3 Subgroups and Additional Analyses

Figure S1 shows subgroup analyses. Hazard ratios in analyses by sex (women: adjusted HR 0.86 [95% CI 0.62–1.18] and men: adjusted HR 0.77 [95% CI 0.62–0.95]) and age group (45–69 years: adjusted HR 0.84 [95% CI 0.67–1.05] and ≥ 70 years: adjusted HR 0.89 [95% CI 0.67–1.16]) were similar to those of the primary analyses. In the additional analysis stratified by time since treatment initiation the adjusted HR was 0.70 for < 2 years, 0.84 for 2 to < 5 years and 0.85 for > 5 years (Table S6). Results by country are shown in Table S7.

### 3.4 Sensitivity Analyses

Table 2 shows sensitivity analyses. Findings remained consistent when using a lag‐time period of 2 years between treatment initiation and the start of follow‐up (adjusted HR 0.84 [95% CI 0.70–1.02]) and when restricting the outcome definition to primary diagnoses in the patient registers (adjusted HR 0.84 [95% CI 0.69–1.02]). Incorporation of calendar time into the propensity score yielded an adjusted HR of 0.82 (95% CI 0.68–0.98), while use of a calendar time‐specific propensity score model resulted in an adjusted HR of 0.84 (95% CI 0.66–1.06). In the analysis excluding and censoring patients at DPP‐4 inhibitor use, the adjusted HR was 0.74 (95% CI 0.60–0.93). Consistency persisted when we excluded patients older than 80 years (adjusted HR, 0.79 [95% CI 0.66–0.94]). When applying a strict new‐user definition, the adjusted HR was 0.85 (95% CI 0.70–1.04); within this cohort, an analysis employing an intention‐to‐treat exposure definition where patients were not censored at treatment switch resulted in an adjusted HR of 0.90 (95% CI 0.74–1.09). The analysis with inverse probability of censoring weighting produced an identical hazard ratio as the primary analysis, adjusted HR 0.81 (0.68–0.96). In the analysis requiring a Parkinson's disease diagnosis at two separate encounters, the adjusted HR was 0.76 (95% CI, 0.63–0.93). The competing risk analysis accounting for death produced a consistent adjusted subdistribution HR of 0.80 (95% CI 0.67–0.95). The total numbers of deaths during follow‐up for each treatment group are provided in Table S8. In the analysis with additional adjustment for glycated haemoglobin, blood pressure, albuminuria, eGFR, body mass index and smoking in the Swedish part of the cohort (patient characteristics are shown in Table S9), the point estimate for the HR was similar to the country‐specific analysis without such adjustment (Table S10).

**TABLE 2:** Results for primary analysis and prespecified sensitivity analyses of the association between use of GLP‐1 receptor agonists and sulfonylureas and risk of incident Parkinson's disease.

Analysis	GLP‐1 receptor agonists			Sulfonylureas			Adjusted hazard ratio (95% CI)^a^
	No. of patients	Events	Incidence rate (events per 10 000 person‐years)	No. of patients	Events	Incidence rate (events per 10 000 person‐years)	
Primary analysis	158 961	290	5.2	188 065	927	8.0	0.81 (0.68–0.96)
Sensitivity analysis							
24 months lag‐time analysis	89 673	215	6.7	154 878	746	9.2	0.84 (0.70–1.02)
Outcome definition restricted to primary diagnoses	158 961	233	4.2	188 065	690	6.0	0.84 (0.69–1.02)
Additional adjustment for calendar time	158 961	290	5.2	188 065	927	8.0	0.82 (0.68–0.98)
Calendar‐time specific propensity score	158 524	290	5.2	181 030	890	7.9	0.84 (0.66–1.06)
Exclusion of patients with previous use of DPP‐4 inhibitors and censoring of patients initiating DPP‐4 inhibitors during follow‐up^b^	99 581	163	4.9	162 072	679	8.2	0.74 (0.60–0.93)
Exclusion of patients > 80 years	151 925	277	5.1	162 285	841	8.1	0.79 (0.66–0.94)
Strict new‐user definition with unrestricted lookback period	125 568	205	4.8	162 534	767	7.6	0.85 (0.70–1.04)
Strict new‐user definition with unrestricted lookback period and ITT exposure criteria	125 568	205	4.8	162 534	812	7.6	0.90 (0.74–1.09)
Inverse probability of censoring weighting analysis	158 961	290	5.2	188 065	927	8.0	0.81 (0.68–0.96)
Outcome definition limited to diagnoses recorded at two separate visits	158 961	222	4.0	188 065	725	6.3	0.76 (0.63–0.93)

Abbreviations: GLP‐1, glucagon‐like peptide‐1; ITT, intention‐to‐treat.

^a^ Adjusted using SMR weighting based on a propensity score that included sociodemographic characteristics, diabetic drug use, co‐morbidities, co‐medications and health care utilization (Table 1).

^b^ 59 380 (37.4%) GLP‐1 receptor agonist users and 25 993 (13.8%) sulfonylurea users were excluded because of previous use of DPP‐4 inhibitors and 54 117 (33.4%) sulfonylurea users and 8252 (8.3%) GLP‐1 receptor agonist users who initiated a DPP‐4 inhibitor during follow‐up were censored.

## 4 Discussion

In this large cohort study using nationwide registers from three countries to include patients seen in routine clinical practice, use of GLP‐1 receptor agonists compared with use of sulfonylureas was associated with a 19% relative risk reduction of incident Parkinson's disease. Study findings were consistent across a range of predefined sensitivity and subgroup analyses.

Clinical trials have shown that GLP‐1 receptor agonists may be beneficial in patients with established Parkinson's disease. A randomized controlled trial evaluating exenatide versus placebo in patients with moderately severe Parkinson's disease demonstrated significantly decreased motor symptoms [10]. Moreover, in a randomized controlled trial including patients with early Parkinson's disease, treatment with lixisenatide resulted in slower progression of motor disability in comparison to placebo [11]. In contrast, in a randomized controlled trial including patients with early, untreated Parkinson's disease, treatment with a pegylated, brain‐penetrant version of exenatide (NLY01) did not affect motor symptom progression [22].

No clinical trials have investigated the effect of GLP‐1 receptor agonists on incident Parkinson's disease, and observational evidence remains limited. In a cohort study based on primary care data in the UK, use of GLP‐1 receptor agonists was associated with a reduced risk of incident Parkinson's disease (incidence rate ratio 0.38, 95% CI 0.17–0.60) in comparison to other non‐insulin glucose‐lowering drugs. However, the study was limited by the lack of a new‐user design, a heterogeneous comparator group and only 17 outcome events [12]. Two subsequent active‐comparator new‐user studies using dipeptidyl peptidase‐4 (DPP‐4) inhibitors as the reference reported directionally consistent findings: a US Medicare study observed a hazard ratio of 0.77 (95% CI 0.63–0.95) based on 143 events, and a Danish cohort study reported an HR of 0.57 (95% CI 0.37–0.85) in its primary on‐treatment analysis [13, 14]. However, the early separation of Kaplan–Meier curves in these studies raises questions regarding a neuroprotective mechanism and could potentially reflect protopathic bias or residual confounding. Observational studies indicate that DPP‐4 inhibitors may themselves be associated with a reduced risk of Parkinson's disease. Consequently, their use as an active comparator may have attenuated the estimated treatment effects [12, 23].

A strength of our study is the use of a robust study design restricting the cohort to new users and the use of a suitable active comparator. Sulfonylureas were selected as the active comparator because both drug classes were recommended as second‐ or third‐line glucose‐lowering agents during the study period, ensuring comparable disease stage and clinical indication at cohort entry. Sulfonylureas act through a mechanism confined to peripheral pancreatic insulin secretion and are regarded as pharmacologically neutral with respect to Parkinson's disease risk [24, 25]. DPP‐4 inhibitors and SGLT‐2 inhibitors were considered less appropriate comparators, as observational data have suggested protective associations with Parkinson's disease risk for both drug classes, which would attenuate any estimated effect of GLP‐1 receptor agonists toward the null [12, 20, 26]. Differential temporal trends in the use of the two drug classes during the study period were addressed through calendar‐time adjustment in sensitivity analyses, with no material change in the estimates (Table 2).

Although additional research is needed to determine whether the observed association reflects a true neuroprotective effect of GLP‐1 receptor agonists, our findings are consistent with the hypothesis that this drug class may reduce the risk of Parkinson's disease and support the rationale for further investigation. In perspective, while widespread use of GLP‐1 receptor agonists for the prevention of Parkinson's disease in the general population is unrealistic because of the relatively low background risk of the disease, future research might target the role of GLP‐1 receptor agonists in specific populations at particularly high risk of Parkinson's disease.

This study also had limitations. First, residual confounding from unmeasured variables remains a potential limitation despite adjustment for a broad range of baseline covariates. Data on HbA1c, BMI and other clinical parameters were unavailable for the full cohort; however, estimates remained consistent in the Swedish subcohort after additional adjustment for these variables, alongside blood pressure, albuminuria, eGFR and smoking status (Table S10). Second, Parkinson's disease has an insidious onset, and the shorter median follow‐up among GLP‐1 receptor agonist users (2.5 years) compared with sulfonylurea users (5.8 years) reflects the increasing uptake of GLP‐1 receptor agonists during the latter part of the study period. Both treatment groups contributed person‐time throughout the entire study period, and the Cox proportional hazards model accounted for time since treatment initiation. Moreover, one quarter of GLP‐1 receptor agonist users were followed for at least 5.2 years, providing sufficient follow‐up time for Parkinson's disease to emerge in a substantial proportion of the exposed cohort. Randomized trials have observed effects on motor symptoms within 12 months of treatment initiation, suggesting that a biologically meaningful signal would be detectable within the available follow‐up window [10, 11]. Third, although high sensitivity and positive predictive values have been observed for diagnoses recorded in Scandinavian health registers, there is a risk of outcome misclassification [27, 28]. A Swedish validation study of the ICD‐10 codes used for Parkinson's disease diagnosis in this study demonstrated a positive predictive value of 71% [29], which increased to 83% when restricting the outcome to primary diagnoses. Fourth, the exposure definition was based on filled prescriptions. Low adherence may have biased the findings toward the null. Fifth, the study was performed in Scandinavia and its generalizability to other populations and health care systems is unknown. Sixth, Protopathic bias may be a concern if prodromal symptoms of Parkinson's disease influenced treatment decisions, leading to differential avoidance of injectable GLP‐1 receptor agonists prior to diagnosis. However, randomized trials have indicated effects on motor symptoms within 12 months of treatment initiation, supporting the decision not to impose a lag‐time in the primary analysis. The sensitivity analysis imposing a 24‐month lag yielded a consistent point estimate (adjusted hazard ratio, 0.84; 95% CI, 0.70–1.02) and the cumulative incidence curves diverged gradually over time rather than separating early after treatment initiation, as would be expected under protopathic bias. Time‐stratified analyses showed some attenuation with longer follow‐up (less than 2 years: HR 0.70; 2 to less than 5 years: HR 0.84; 5 or more years: HR 0.85), though a directionally consistent association persisted across all strata (Table S6). The modest attenuation beyond 5 years may reflect the intention‐to‐treat nature of the analysis, as accumulating treatment discontinuation over time would be expected to attenuate estimates toward the null independent of any true neuroprotective effect. Seventh, patients who initiated liraglutide contributed with most of the follow‐up time for GLP‐1 receptor agonists. Therefore, the results of this study mainly apply to this specific drug.

## 5 Conclusion

In this large cohort study, use of GLP‐1 receptor agonists compared with use of sulfonylureas was associated with a lower risk of incident Parkinson's disease. These results support a potential neuroprotective role of GLP‐1 receptor agonists and warrant further investigation.

## Author Contributions

Concept and design: Arvid Engström, Henrik Svanström, Peter Ueda and Björn Pasternak. Acquisition, analysis or interpretation of data: all authors. Drafting of the manuscript: Arvid Engström, Peter Ueda and Björn Pasternak. Critical revision of the manuscript for important intellectual content: all authors. Statistical analysis: Henrik Svanström. Obtained funding: Peter Ueda and Björn Pasternak. Study supervision: Peter Ueda and Björn Pasternak. Drs. Henrik Svanström and Björn Pasternak are the guarantors of this work and, as such, had full access to all the data in the study and take responsibility for the integrity of the data and the accuracy of the data analysis.

## Funding

The study was supported by grants from the Swedish Research Council, Region Stockholm (ALF) and Dr. Margaretha Nilsson's Foundation for Medical Research. Dr. Björn Pasternak was supported by a consolidator investigator grant from Karolinska Institutet. Dr. Peter Ueda was supported by a grant from the Strategic Research Area Epidemiology programme and a Faculty Funded Career Position at Karolinska Institutet. Prof. Anders Hviid was supported by an investigator grant and holds a faculty recruitment grant from the Novo Nordisk Foundation; by an investigator grant from the Lundbeck Foundation; and by a research grant from the Independent Research Foundation Denmark. Dr. Björn Eliasson was supported by Konung Gustaf V:s och Drottning Victorias Frimurarestiftelse. Prof Mads Melbye was supported by a research grant from the Independent Research Foundation Denmark and a grant from the Danish Cancer Society.

## Disclosure

The funding sources had no role in the design and conduct of the study; collection, management, analysis and interpretation of the data; preparation, review or approval of the manuscript; and decision to submit the manuscript for publication.

## Conflicts of Interest

All authors have completed the ICMJE uniform disclosure form at www.icmje.org/coi_disclosure.pdf and have the following declarations. Dr. Christian Jonasson is an employee of NordicRWE. Dr. Björn Eliasson reports personal fees from Amgen, AstraZeneca, Boehringer Ingelheim, Eli Lilly, Merck Sharp & Dohme, Mundipharma, Navamedic, Novo Nordisk and Sanofi outside the submitted work. Dr. Henrik Svanström is a former employee of IQVIA. Prof. Anders Hviid is a scientific advisory board member of VAC4EU. The other authors declare no conflicts of interest.

## Supporting information


**Table S1:** ATC‐codes and estimated days of supply per unit by type of GLP‐1 receptor agonists and sulfonylureas.
**Table S2:** ICD10 and procedure codes for exclusion criteria.
**Table S3:** Covariates for propensity score.
**Table S4:** Variable definitions for the analyses using data from the National Diabetes Register in Sweden.
**Table S5:** Absolute incidence rate differences for incident Parkinson's disease at prespecified time intervals.
**Figure S1:** Subgroup analyses of incident Parkinson's disease among users of GLP‐1 receptor agonists compared with users of sulfonylureas.
**Table S6:** Additional analysis of incident Parkinson's disease stratified by time since treatment initiation.
**Table S7:** Analyses of incident Parkinson's disease by country.
**Table S8:** Total number of deaths during follow‐up.
**Table S9:** Distribution of variables from the Swedish National Diabetes Register in the Swedish part of cohort.
**Table S10:** Sensitivity analysis including additional variables in the Swedish part of the cohort.

## Data Availability

No additional data available. The data analysed in this study were based on Scandinavian nationwide register. Individual level data from the registers can only be accessed through secure servers and only export of aggregated data, as presented in research articles, is allowed. Permission to access data can be made only after fulfilling specific requirements to safeguard the anonymity of the study participants and other data safety issues. For these reasons, data cannot be made generally available.
