# Supplementary material for: Use of Glucagon‐Like Peptide‐1 Receptor Agonists and Risk of Parkinson's Disease: Scandinavian Cohort Study
Source: Diabetes Obes Metab. 2026 Apr 17;28(7):5767–78. doi: 10.1111/dom.70760 (PMC13243990; doi:10.1111/dom.70760)
Supplement: Supplementary file 1 — Table S1: ATC‐codes and estimated days of supply per unit by type of GLP‐1 receptor agonists and sulfonylureas. Table S2: ICD10 and procedure codes for exclusion criteria. Table S3: Covariates for propensity score. Table S4: Variable definitions for the analyses using data from the National Diabetes Register in Sweden. Table S5: Absolute incidence rate differences for incident Parkinson's disease at prespecified time intervals. Figure S1: Subgroup analyses of incident Parkinson's disease among users of GLP‐1 receptor agonists compared with users of sulfonylureas. Table S6: Additional analysis of incident Parkinson's disease stratified by time since treatment initiation. Table S7: Analyses of incident Parkinson's disease by country. Table S8: Total number of deaths during follow‐up. Table S9: Distribution of variables from the Swedish National Diabetes Register in the Swedish part of cohort. Table S10: Sensitivity analysis including additional variables in the Swedish part of the cohort. [file DOM-28-5767-s001.docx]

**ONLINE-ONLY SUPPLEMENTAL MATERIAL**

**Use of Glucagon-Like Peptide-1 Receptor Agonists and Risk of Parkinson’s Disease: Scandinavian Cohort Study**

Engström et al.

| **TABLE OF CONTENTS** | **PAGE** |
| --- | --- |
| **Data sources** | 2 |
| **Supplemental table 1.**  ATC-codes and estimated days of supply per unit by type of GLP-1 receptor agonists and sulfonylureas. | 3 |
| **Supplemental table 2.** ICD10 and procedure codes for exclusion criteria. | 4 |
| **Supplemental table 3.** Covariates for propensity score. | 6 |
| **Supplemental table 4.**  Variable definitions for the analyses using data from the National Diabetes Register in Sweden. | 9 |
| **Supplemental table 5.**  Absolute incidence rate differences for incident Parkinson’s Disease at prespecified time intervals | 10 |
| **Supplemental figure 1.** Subgroup analyses of incident Parkinson’s disease among users of GLP-1 receptor agonists compared with users of sulfonylureas. | 11 |
| **Supplemental table 6.**  Additional analysis of incident Parkinson’s disease stratified by time since treatment initiation. | 12 |
| **Supplemental table 7.**  Analyses of incident Parkinson’s disease by country. | 13 |
| **Supplemental table 8.** Total number of deaths during follow-up. | 14 |
| **Supplemental table 8.**  Distribution of variables from the Swedish National Diabetes Register in the Swedish part of cohort. | 15 |
| **Supplemental table 9.**  Sensitivity analysis including additional variables in the Swedish part of the cohort. | 17 |

**Data sources**

Data on filled prescriptions were obtained from the national prescription registers in Sweden^1^, Denmark^2^ and Norway^3^. The registers contain individual-level data on all drug prescriptions filled at all pharmacies in the country since July 2005, in Sweden, 1995 in Denmark, and 2004 in Norway and include the anatomical therapeutic chemical (ATC) code of the dispensed drug, information about the amount of drug dispensed and the date when the prescription was filled.

The national patient registers comprise individual-level data on outpatient and emergency department visits and inpatient admissions to all hospitals in the country.^4–6^ In the present study, these registers were used to obtain information about history of disease at cohort entry for each patient and the outcome events during the study period by using physician-assigned procedure codes, and diagnoses according to the International Classification of Diseases, tenth revision (ICD10).

The population registers were used to obtain information on age, sex, country of birth, migration status, civil status (Norway) and vital status of individual patients^7–9^ Information about patients’ educational level and civil status were obtained from Statistics Denmark and Statistics Sweden.

The National Diabetes Register includes data on risk factors of cardiovascular disease and diabetes complications among patients with type 1 or type 2 diabetes in Sweden. Data are collected by trained nurses and physicians during patient visits to primary care and outpatient clinics nationwide. At present, over 90% of all patients receiving drugs for diabetes in Sweden are included, with this number having increased during the past years.^10^ For the Swedish part of the cohort, we used this register to obtain data on glycated hemoglobin, blood pressure, albuminuria, estimated glomerular filtration rate (eGFR), body-mass index and smoking.

The Danish Register of Laboratory Results for Research collects nationwide data from routine biomarkers from general practitioners and hospital encounters.^11^ Data collection started between 2013 and 2015 depending on region. From this register, we obtained information about glycated haemoglobin, albuminuria and eGFR for the Danish part of the cohort.

The personal identification number assigned to all inhabitants in the three countries enabled linkage of individual-level information across data sources.

| **Supplemental table 1.** ATC-codes and estimated days of supply per unit by type of GLP-1 receptor agonists and sulfonylureas. | | | | |
| --- | --- | --- | --- | --- |
| **Category** | **ATC definition** | **Estimated days of supply Sweden** | **Estimated days of supply Denmark** | **Estimated days of supply Norway** |
| **GLP-1 receptor agonists** | A10BJ01, A10BJ02 (not including saxenda -product no. 131577, 395175, 164108, 439932 and 575140 in Denmark and Norway, 034982 in Norway and  513490, 141823, 439932, 026334 or 471462 in Sweden), A10BJ03, A10BJ05, A10BJ06, A10AE54, A10AE56 | A10BJ01; 5 or 10 microgram = 0.5 per dose  A10BJ01; 2 mg = 7.0 per dose  A10BJ02 = 5.0 per ml  A10BJ03 = 1.0 per dose  A10BJ05 = 7.0 per dose  A10BJ06 = 7.0 per dose  A10AE54 = 2.5 per ml  A10AE56 = 3.0 per ml | A10BJ01; 5 or 10 microgram = 0.5 per dose  A10BJ01; 2 mg = 7.0 per dose  A10BJ02 = 15.0 per pen  A10BJ03 = 14.0 per pen  A10BJ05 = 7.0 per dose  A10BJ06 = 28.0 per pen  A10AE54 = 2.5 per ml  A10AE56 = not available | A10BJ01; 5 or 10 microgram = 0.5 per dose  A10BJ01; 2 mg = 7.0 per pen  A10BJ02 = 5.0 per ml  A10BJ03 = 1.0 per dose  A10BJ05 = 7.0 per pen  A10BJ06 = 7.0 per dose  A10AE54 = 2.5 per ml  A10AE56 = 3.0 per ml |
| **Sulfonylureas** | A10BB  A10BD01  A10BD02  A10BD04  A10BD06 | …… | …… | …… |

Abbreviations: GLP-1, glucagon-like peptide-1

| **Supplemental table 2.** ICD10 and procedure codes for exclusion criteria. | | |
| --- | --- | --- |
| **Category** | **Codes (ICD-10, procedure, or ATC)** | **Data source / Type of diagnosis / type of hospital contact** |
| Initiation of both study drugs on the same day | n.a. | Prescribed drug register |
| End stage illness (severe malnutrition, cachexia, dementia, coma) at any time before index date^a^ | ICD-10: E40-E43, F00-F03, G30, R40.2^b^, R64 ATC: N06D | Patient register, any position, any type hospital contact; prescribed drug register |
| Drug misuse within last year | ICD-10: F11-F16, F18, F19, R78.1-R78.5^b^ T40^b^ ATC: N07BC | Patient register, any position, any type hospital contact; prescribed drug register |
| No specialist care contact or prescription drug in last year prior to the index date | n.a. | Patient register, prescribed drug register |
| Dialysis or renal transplantation at any time before index date^a^ | ICD-10: Z49, Z94.0, Z99.2 Procedure: KAS  Additional procedure codes:  Sweden: DR012, DR013, DR014, DR015, DR016, DR023, DR024, DR055, DR056, DR060, DR061  Denmark: (B)JFD, (B)JFZ  Norway: A0093, A0094, JAGD30, JAGD31, JAGD32, JAGD50, JAK10, PHGX00, PHGX05, RXGD05, RXGD20, RXGD25, TJA33 | Patient register, any position |
| Major pancreatic disease (chronic pancreatitis [defined by pancreatic enzyme substitution prescription within last year or diagnosis at any time before index date], pancreatic cancer, major pancreatic surgery at any time before index date^a^) | ICD-10: C25, K86.0, K86.1  Procedure: JLC, JLE ATC: A09AA02 | Patient register, any position, any type hospital contact; prescribed drug register |
| Liraglutide with obesity indication (Saxenda) at any time before index date^c^ | A10BJ02 with product number 131577, 395175 164108, 439932 or 575140 in Denmark and Norway, 034982 in Norway and 513490, 141823, 439932, 026334 or 471462 in Sweden. | Prescribed drug register |
| History of Parkinson’s disease, secondary parkinsonism or Lewy body dementia at any time before index date | G20-G23, G31.8 | Patient register, any position, any type of contact |
| Filled prescription of anti-parkinson drugs within last year | ATC:N04 anti-parkinson drugs | Prescribed drug register |
| Abbreviations: ICD, International Classification of Diseases; ATC, Anatomical Therapeutic Chemical  ^a^ 5-year lookback in Sweden and Denmark; 2-year look-back in Norway ^b^ Not available in the Norwegian dataset.  ^c^  Any time defined as ever during time period drug has been available | | |

| **Supplemental table 3.** Covariates for propensity score. | |
| --- | --- |
| **Sociodemographic characteristics** | **ICD/categories** |
| Sex | Women/men |
| Age | Cubic splines |
| Place of birth | Scandinavia; Rest of Europe; Outside Europe, Missing |
| Living with partner | Yes/no |
| Education ^a^ | Primary school and high school; vocational or short-term tertiary education; medium or long tertiary education; missing; Norway |
| **Medical history (5 yr look-back in Sweden and Denmark; 2 year look-back in Norway)** | *ICD-10 code and procedure code* |
| Ischemic heart disease | ICD-10: I20-I25, I 11 (not I110).  Procedure: F (except FPFE, FPGX), DF020 |
| Heart failure/cardiomyopathy | ICD-10: I50, I110, I130, I132, I42, I43, J81 |
| Stroke/cerebrovascular disease | ICD-10: I60-I69, G45 (excl G454), G46, I69 |
| Head trauma | S020, S021, S027-S029, S060-S071 |
| Other neurological disorders | G01-G19, G24-G44, G454, G47-G99 |
| Arrhythmia | ICD-10: I44-I49 |
| Peripheral arterial disease (including amputation) | ICD-10: I65, I70, I72, I73, I74, I77, K550, K551, E115, E145, E135  Procedure: NFQ ,NGQ, NHQ |
| Kidney disease | ICD-10:  E112, E132, E142, I120, I131, I132, N00-08, N10-N23, N25-N29 |
| Diabetes complications | ICD-10: E110, E111, E113, E114, E116, E117, E118, E130, E131, E133, E134, E136, E137, E138, E140, E141, E143, E144, E146, E147, E148, E160, E161, E162, G990, G590, G632, H280, H358, H360, M142, M146, M908, L984  Procedure: CKC10, CKC12, CKC15, CKD65 |
| COPD | ICD-10: J44 |
| Other lung disease | ICD-10: I27, J84, R092, E662, Z99, J40-J43, J45-J47, J60-J69, J70,^b^ J92, J96, J982, J983  Procedure: GBB |
| Venous thromboembolism | ICD-10: I26, I80 (except I80.0), I81, I820, I822-I829 |
| Cancer (excl skin cancer) | ICD-10: C00-C42, C45-C97 |
| Melanoma | ICD-10: C43 |
| Liver disease | ICD-10: B18, I850, I859, I982, K70-K77 |
| Thyroid disease | ICD-10: E012 E018 E032 E038 E040 E041 E042 E049 E890 E06 |
| Osteoporosis | ICD10: M80-M82 ATC: M05BA, M05BB M05BX04 M05BX06 |
| Alcohol-related disorders | ICD-10: E244, F101-F109, G312, G621, G721, I426, K292, K70, K852, X65^b^  ATC: N07BB |
| **Medical history (1 yr look-back)** | *ICD-10 code and procedure code* |
| Fracture in previous year | ICD-10: S02 (except S025), S12, S22, S32, S42, S52, S62, S72, S82, S92, T02, T08, T10, T12, M484, M485, M843 |
| **Prescription-drug use in previous yr** | *ATC code* |
| ACE-inhibitor or ARB | C09A-D |
| Calcium channel blockers | C08C, C08D |
| Spironolactone | C03DA01 |
| Loop diuretic | C03C, C03EB |
| Other diuretic | C03A, C03B, C03D, C03EA |
| Beta-blocker | C07 |
| Other cardiovascular drugs | C01AA05, C01DA |
| Antiarrhythmic drug | C01B |
| Platelet inhibitor | B01AC |
| Anticoagulant | B01AA, B01AE07, B01AF, B01AX05 |
| Lipid lowering drug | C10 |
| Lithium | N05AN |
| Typical Antipsychotic | N05AA, N05AB, N05AC, N05AD, N05AE, N05AF, N05AG |
| Atypical antipsychotic | N05AH, N05AX |
| Antidepressant | N06A |
| Anxiolytic, hypnotic or sedative | N05B, N05C |
| Beta-2 agonist inhalant | R03AC |
| Anticholinergic inhalant | R03BB |
| Glucocorticoid inhalant | R03BA, R03AK |
| Oral glucocorticoid | H02AB |
| Opiate | N02A |
| Antiepileptic | N03A |
| Urate-lowering drug | M04A |
| **Diabetes drugs in the last 6 months** |  |
| Metformin | A10BA02, A10BD02, A10BD03, A10BD05, A10BD07, A10BD08, A10BD10, A10BD11, A10BD13, A10BD14, A10BD15, A10BD16, A10BD20 |
| Other 2^nd^ line antidiabetics | SGLT2 inhibitors A10BK01, A10BK02, A10BK03, A10BK04, A10BD15, A10BD16, A10BD19, A10BD20, A10BD21, A10BD23, A10BD24, A10BD25  DPP4 inhibitors A10BH01, A10BH02, A10BH03, A10BH04, A10BH05, A10BD07, A10BD08, A10BD09, A10BD10, A10BD11, A10BD13, A10BD19, A10BD21, A10BD24, A10BD25  Insulin A10AB, A10AC, A10AD, A10AE  Glitazones, glinides, acarbose A10BF01, A10BG, A10BD03, A10BD04, A10BD05, A10BD06, A10BD09, A10BD14, A10BX |
| **Health care utilization in previous year** |  |
| No. of drugs used ^c^ | <5, 6-10, 11-15, >15 |
| Hospitalization due to neurological causes | G00-G99 (primary position) |
| Hospitalization due to non-neurological causes | Not G00-G99 (Primary position) |
| Outpatient contact due to neurological causes | G00-G99 (primary position) |
| Outpatient contact due to non-neurological causes | Not G00-G99 (primary position) |
| Abbreviations: ACE-I, angiotensin converting enzyme inhibitor; ARB, angiotensin receptor blocker; COPD, chronic obstructive pulmonary disease; DDP4, dipeptidyl peptidase 4; SGLT2, sodium-glucose cotransporter-2; NSAID, non-steroidal anti-inflammatory drug.  ^a^ Information about education was not available in Norway.  ^b^ Not available in Norway.  ^c^ In the Norwegian dataset, the number of drugs used for the ATC-codes included as variables in the propensity score were used. In Sweden and Denmark, all ATC-codes were used. | |

| **Supplemental table 4.** Variable definitions for the analyses using data from the National Diabetes Register in Sweden. | | |
| --- | --- | --- |
| **Variable** | **Categorization** | **% missing values (Sweden)^a^** |
| HbA1c (mmol/mol) | ≤52; 53-62; 63-72; 73-82; ≥83 | 40 |
| Albuminuria | Normalbuminuria; microalbuminuria; macroalbuminuria | 38 |
| eGFR (ml/min) | <30; 30-59; 60-89; ≥90 | 27 |
| Blood pressure | *Normotension:*  SBP <140 mmHg AND DBP <90 mmHg  *Stage 1 hypertension:*  SBP ≥140 to <160 mmHg OR DBP: ≥90 to <100mmHg  *Stage 2 hypertension:*  SBP ≥160 mmHg OR DBP: ≥100 mmHg | 25 |
| Body-mass index (kg/m2) | Normal weight: <25  Overweight: ≥25 to <30  Obese class I: ≥30 to <35  Obese class II: ≥35 | 30 |
| Current smoking | Yes/no | 31 |

Abbreviations: SBP: systolic blood pressure; DBP: diastolic blood pressure; eGFR: estimated glomerular filtration rate.

a Missing values in the Swedish part of the cohort of GLP-1 receptor agonist users and sulfonylureas users.

| **Supplemental table 5.**  Absolute incidence rate differences for incident Parkinson’s Disease at prespecified time intervals. | | | |
| --- | --- | --- | --- |
|  | **GLP-1 receptor agonists** | **Sulfonylureas** |  |
| **Follow-up** | **Incidence rate (events per 10 000 person- years)** | **Incidence rate (events per 10 000 person- years)** | **Adjusted rate difference per 10 000 person-years (95% CI)^a^** |
| 3 years | 3.4 | 5.5 | -1.5 (-2.8 to -0.1) |
| 5 years | 4.2 | 6.5 | -1.5 (-2.7 to -0.3) |
| 7 years | 4.8 | 7.2 | -1.7 (-2.8 to -0.6) |
| Total | 5.2 | 8.0 | -2.1 (-3.2 to -1.0) |
| Abbreviations: GLP-1, glucagon-like peptide-1  ^a^ Adjusted using SMR weighting based on a propensity score that included sociodemographic characteristics, diabetic drug use, co-morbidities, co-medications and health care utilization (Table 1). | | | |

| **Supplemental figure 1.** Subgroup analyses of incident Parkinson’s disease among users of GLP-1 receptor agonists compared with users of sulfonylureas. | | | | | | |  | |
| --- | --- | --- | --- | --- | --- | --- | --- | --- |
| **Table 3.** Subgroup and additional analyses of incident AF among users of SGLT2 inhibitors compared with users of GLP-1 receptor agonists | **GLP-1 receptor agonists** | | **Sulfonylureas** |  |  |  |  |  |
|  | **No. of patients** | **Events/events per 10 000 person years** | **No. of patients** | **Events/events per 10 000 person years** | **Adjusted hazard ratio (95% CI)^a^** | | **Adjusted rate difference per 10 000 patient years (95% CI) ^a^** |  |
| *Total population* | 158 961 | 290/5.2 | 188 065 | 927/8.0 | 0.81 (0.68 to 0.96) | 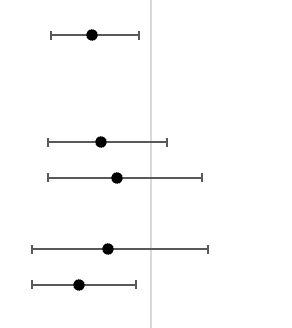 | -2.1 (-3.2 to -1.0) |  |
| **Subgroup analyses** | | |  |  |  |  |  |  |
| *Age* | | |  |  |  |  |  |  |
| 45-69 years | 117 870 | 187/4.1 | 115 334 | 434/5.7 | 0.84 (0.67 to 1.05) |  | -1.6 (-2.7 to -0.4) |  |
| ≥70 years | 41 081 | 103/9.6 | 72 670 | 494/12.3 | 0.89 (0.67 to 1.16) |  | -3.5 (-6.4 to -0.5) |  |
| *Sex* | | |  |  |  |  |  |  |
| women | 67 981 | 90/3.8 | 79 922 | 281/5.7 | 0.86 (0.62 to 1.18) |  | -1.5 (-2.9 to -0.1) |  |
| men | 90 956 | 200/6.2 | 107 793 | 645/9.7 | 0.77 (0.62 to 0.95) |  | -2.7 (-4.3 to -1.2) |  |
|  |  |  |  |  |  |  |  |  |
|  |  |  |  |  |  | 0.5 1 1.5 |  |  |
| Abbreviations: GLP-1, glucagon-like peptide-1  ^a^ Adjusted using SMR weighting based on a propensity score that included sociodemographic characteristics, diabetic drug use, co-morbidities, co-medications and health care utilization (Table 1). | | | | | | |  |  |

| **Supplemental table 6.**  Additional analysis of incident Parkinson’s disease stratified by time since treatment initiation | | | | | | | |
| --- | --- | --- | --- | --- | --- | --- | --- |
|  | **GLP-1 receptor agonists** | | | **Sulfonylureas** | | |  |
| **Time period** | **No. of patients** | **Events** | **Incidence rate (events per 10 000 person- years)** | **No. of patients** | **Events** | **Incidence rate (events per 10 000 person- years)** | **Adjusted hazard ratio (95% CI)^a^** |
| <2 years | 158961 | 75 | 3.1 | 188065 | 181 | 5.3 | 0.70 (0.48 to 1.02) |
| 2 to <5 years | 89673 | 106 | 5.7 | 154878 | 298 | 7.6 | 0.85 (0.65 to 1.12) |
| >5 years | 41812 | 109 | 8.2 | 106129 | 448 | 10.6 | 0.85 (0.65 to 1.10) |
| Abbreviations: GLP-1, glucagon-like peptide-1  ^a^ Adjusted using SMR weighting based on a propensity score that included sociodemographic characteristics, diabetic drug use, co-morbidities, co-medications and health care utilization (Table 1). | | | | | | | |

| **Supplemental table 7.** Analyses of incident Parkinson’s disease by country. | | | | | | | |
| --- | --- | --- | --- | --- | --- | --- | --- |
|  | **GLP-1 receptor agonists** | | | **Sulfonylureas** | | |  |
| **Country** | **No. of patients** | **Events** | **Incidence rate (events per 10 000 person- years)** | **No. of patients** | **Events** | **Incidence rate (events per 10 000 person- years)** | **Adjusted hazard ratio (95% CI)^a^** |
| Denmark | 61 343 | 115 | 5.0 | 56 887 | 297 | 8.1 | 0.71 (0.53 to 0.96) |
| Norway | 12 432 | 19 | 4.8 | 30 047 | 118 | 9.0 | 0.51 (0.29 to 0.88) |
| Sweden | 85 186 | 156 | 5.4 | 101 131 | 512 | 7.8 | 0.92 (0.73 to 1.17) |
| Abbreviations: GLP-1, glucagon-like peptide-1  ^a^ Adjusted using SMR weighting based on a propensity score that included sociodemographic characteristics, diabetic drug use, co-morbidities, co-medications and health care utilization (Table 1). | | | | | | | |

|  |
| --- |
| \| **Supplemental table 8.** Total number of deaths during follow-up. \| \| \| \| \| --- \| --- \| --- \| --- \| \| **Exposure status** \| **No. of patients** \| **Total person-years of follow up** \| **Total number of deaths** \| \| GLP-1 receptor agonists \| 158 961 \| 559 979 \| 12 231 \| \| Sulfonylureas \| 188 065 \| 1 156 732 \| 53 736 \| \| Abbreviations: GLP-1, glucagon-like peptide-1 \| \| \| \| |

| **Supplemental table 9.** Distribution of variables from the Swedish National Diabetes Register in the Swedish part of cohort. All values are n (%). | | |
| --- | --- | --- |
|  | **GLP-1 receptor agonists** | **Sulfonylureas** |
| **Blood pressure (mmHg)** |  |  |
| SBP < 140 and DBP < 90 | 36 764 (43) | 33 135 (33) |
| SBP 140-159 or DBP 90-99 | 21 038 (25) | 20 646 (20) |
| SBP ≥160 or DBP ≥100 | 6 144 (7) | 7 138 (7) |
| Missing | 21 244 (25) | 40 340 (40) |
| **HbA1c (mmol/mol)** |  |  |
| ≤52 | 7 257 (9) | 6 817 (7) |
| 53-62 | 13 692 (16) | 15 099 (15) |
| 63-72 | 13 010 (15) | 10 519 (10) |
| 73-82 | 8 015 (9) | 5 059 (5) |
| ≥83 | 8 892 (10) | 4 881 (5) |
| Missing | 34 324 (40) | 58 884 (58) |
| **Body mass index (kg/m2)** |  |  |
| <25 | 2 888 (3) | 8 412 (8) |
| 25-29 | 14 914 (18) | 22 171 (22) |
| 30-34 | 21 177 (25) | 16 611 (16) |
| ≥35 | 20 934 (25) | 8 825 (9) |
| Missing | 25 277 (30) | 45 240 (45) |
| **Albuminuria** |  |  |
| Normalbuminuria | 37 798 (44) | 35 733 (35) |
| Microalbuminuria | 11 421 (13) | 8 861 (9) |
| Macroalbuminuria | 3 279 (4) | 2 623 (3) |
| Missing | 32 692 (38) | 54 042 (53) |
| **eGFR (ml/min)** |  |  |
| ≥90 | 25 230 (30) | 22 304 (22) |
| 60-89 | 25 247 (30) | 25 547 (25) |
| <60 | 11 550 (14) | 9 605 (9) |
| Missing | 23 163 (27) | 43 803 (43) |
| **Current smoking** |  |  |
| No | 50 994 (60) | 46 725 (46) |
| Yes | 7 788 (9) | 8 557 (8) |
| Missing | 26 408 (31) | 45 977 (45) |
| Abbreviations: SBP, systolic blood pressure; DBP, diastolic blood pressure; eGFR, estimated glomerular filtration rate; GLP-1, glucagon-like peptide | | |

| **Supplemental table 10.** Sensitivity analysis including additional variables in the Swedish part of the cohort. | | | | | | | | | |
| --- | --- | --- | --- | --- | --- | --- | --- | --- | --- |
|  | **GLP-1 receptor agonists** | | | **Sulfonylureas** | | |  |  |  |
| **Outcome** | **No. of patients** | **Events** | **incidence rate (events per 10 000 person- years)** | **No. of patients** | **Events** | **incidence rate (events per 10 000 person- years)** | **Adjusted hazard ratio (95% CI) ^a^** | **Adjusted for additional variables**  **hazard ratio (95% CI)^b^** |  |
| Incident Parkinson’s disease | 85 160 | 156 | 5.4 | 101 156 | 511 | 7.7 | 0.92 (0.73 to 1.17) | 0.95 (0.74 to 1.21) |  |
| Abbreviations: GLP-1, glucagon-like peptide-1 | | | | | | | | |  |
| ^a^ Adjusted using SMR weighting based on a propensity score that included sociodemographic characteristics, diabetic drug use, co-morbidities, co-medications and health care utilization (Table 1). | | | | | | | | |  |
| ^b^ In addition to the variables presented in Table 1, this analysis used a propensity score including additional variables, including glycated hemoglobin, blood pressure, albuminuria, estimated glomerular filtration rate, body mass index, and smoking | | | | | | | | |  |

|  |
| --- |

**References**

1. Wettermark B, Hammar N, Fored M, Leimanis A, Olausson PO, Bergman U, et al. The new Swedish Prescribed Drug Register—Opportunities for pharmacoepidemiological research and experience from the first six months. *Pharmacoepidemiology and Drug Safety*. 2007;16(7):726–735.

2. Pottegård A, Schmidt SAJ, Wallach-Kildemoes H, Sørensen HT, Hallas J, Schmidt M. Data Resource Profile: The Danish National Prescription Registry. *International Journal of Epidemiology.* 2017;46(3):798–798f.

3. Norwegian Prescription Database - NIPH n.d. https://www.fhi.no/en/hn/health-registries/norpd/norwegian-prescription-database/ (accessed June 7, 2024).

4. Ludvigsson JF, Andersson E, Ekbom A, Feychting M, Kim J-L, Reuterwall C, et al. External review and validation of the Swedish national inpatient register. *BMC Public Health*. 2011;11:450.

5. Schmidt M, Schmidt SAJ, Sandegaard JL, Ehrenstein V, Pedersen L, Sørensen HT. The Danish National Patient Registry: a review of content, data quality, and research potential. *Clinical Epidemiology*. 2015;7:449–490.

6. Overview of the national health registries - NIPH n.d. https://www.fhi.no/en/more/access-to-data/about-the-national-health-registries2/ (accessed June 7, 2024).

7. Ludvigsson JF, Almqvist C, Bonamy A-KE, Ljung R, Michaëlsson K, Neovius M, et al. Registers of the Swedish total population and their use in medical research. *European Journal of Epidemiology*. 2016;31(2):125–136.

8. Schmidt M, Pedersen L, Sørensen HT. The Danish Civil Registration System as a tool in epidemiology. *European Journal of Epidemiology*. 2014; 29:541–549.

9. National Population Register - The Norwegian Tax Administration n.d. https://www.skatteetaten.no/en/person/national-registry/ (accessed June 7, 2024).

10. Eeg-Olofsson K, Åkesson K, Thorén A et al. National Diabetes Register. Annual Report 2023. https://ndr.registercentrum.se/nyheter/arsrapport-ndr-2023-ars-data (accessed June 7, 2024).

11. Arendt JFH, Hansen AT, Ladefoged SA, Sørensen HT, Pedersen L, Adelborg K. Existing Data Sources in Clinical Epidemiology: Laboratory Information System Databases in Denmark. *Clinical Epidemiology*. 2020;12:469-475.
